# Clinical and structural insights into concurrent EGFR and MET exon 14 skipping mutations in NSCLC: a multi-center series

**DOI:** 10.1186/s40364-025-00864-1

**Published:** 2025-11-28

**Authors:** Lili Shen, Hongyu Deng, Hongyan Liu, Xiaoqiang Huang, Qingming Jiang, Kaihua Liu

**Affiliations:** 1 Department of Pathology, Chongqing University Cancer Hospital, Chongqing, 400030 China; 2 Chongqing Key Laboratory for Intelligent Oncology in Breast Cancer, Chongqing University Cancer Hospital, Chongqing, China; 3 The Affiliated Cancer Hospital of Xiangya School of Medicine, Central South university/Hunan Cancer Hospital, Changsha, Hunan China; 4 Affiliated Hospital of Zunyi Medical University, Zunyi, Guizhou China; 5 Department of Internal Medicine, University of Michigan, Ann Arbor, MI 48109 USA; 6 Nanjing Geneseeq Technology Inc., Nanjing, Jiangsu 210029 China

**Keywords:** Lung cancer, NSCLC, *EGFR*, *MET* exon 14 skipping, *MET* amplification

## Abstract

The co-occurrence of epidermal growth-factor receptor *(EGFR)* mutation and *de novo* or acquired *MET* exon 14 skipping (*MET*ex14) mutation in non-small-cell lung cancer (NSCLC) is extraordinarily rare. No more than five cases across multiple studies have been well documented. Here, we report the largest *EGFR*–*MET*ex14 co-mutation series to date-seven tumours diagnosed between 2019 and 2024 at three Chinese tertiary hospitals—and compare them with 12 *MET*ex14-only and 709 *EGFR*-only cohorts. Co-mutated tumours were dominated by *EGFR*-L858R (71%) and frequently harboured high-level *MET* amplification (57%). Four co-mutations emerged as resistance after first- or third-generation *EGFR* tyrosine-kinase inhibitors (TKIs), with a median post-acquisition progression-free survival (PFS) of 1.5 months and an overall survival (OS) of less than 6 months. Three *de novo* tumours exhibited heterogeneous but occasionally durable control. Building on prior evidence that the cytoplasmic kinase domains of EGFR and MET physically interact, we computationally modelled their domain interactions. Compared to EGFR-19del, EGFR-L858R was predicted to have stronger binding affinity to MET, which may contribute to its clinical enrichment. The poor outcomes associated with concurrent EGFR–MET activation likely reflect the enhanced potential of EGFR-L858R to engage MET signaling and may lead to resistance to EGFR-TKIs even without overt MET amplification. In contrast, EGFR-19del showed weaker MET interaction and may retain EGFR-TKI sensitivity unless MET was secondarily upregulated or amplified. These findings highlight the need for routine molecular re-profiling at progression and support prospective trials of dual EGFR/MET blockade administered upfront in *de novo* co-alteration or at resistance with acquired MET activation.

**Supplementary information:**

The online version contains supplementary material available at 10.1186/s40364-025-00864-1.

**To the editor** The concurrent occurrence of *EGFR* mutations and *MET* exon 14 skipping alteration (METex14) is exceptionally rare. To our knowledge, no more than five well-documented cases have been reported [1, 2]. We here report the largest clinicopathological series to date-seven non-small-cell lung cancers (NSCLCs) collected between January 2019 and January 2024 at three Chinese tertiary (Grade A) hospitals (Chongqing University Cancer Hospital, Hunan Cancer Hospital, and the Affiliated Hospital of Zunyi Medical University). Molecular profiling was performed using next-generation sequencing (NGS) with 14- and 196-gene panels (Supplementary Methods). Four patients acquired *MET*ex14 at progression on first- or third-generation EGFR tyrosine-kinase inhibitors (TKIs), while three patients harbored *EGFR*-*MET*ex14 co-mutations *de novo*.

**Baseline characteristics** As shown in Table 1, the *EGFR*-*MET*ex14 cohort (*n* = 7) showed higher proportions of men (57.1% vs. 50.0% for *MET*ex14-only, 39.2% for *EGFR*-only), stage III-IV disease (85.7% vs. 75.0%, 60.8%), and smoking history (42.9% vs. 33.3%, 29.8%). However, such differences did not reach statistical significance, likely due to small sample size.

**Table 1:** Baseline demographics and tumor characteristics of NSCLC patients with *EGFR*-*MET*ex14 co-mutations, *MET*ex14-only, or *EGFR*-only mutations

	*EGFR***-METex14** (N = 7)	*MET***ex14** (N = 12)	EGFR (N = 709)
**Gender, n (%)**			
Female	3 (42.9%)	6 (50.0%)	431 (60.8%)
Male	4 (57.1%)	6 (50.0%)	278 (39.2%)
**Age (Years)**			
Mean (SD)	62.7 (11.8)	65.2 (12.2)	60.2 (11.0)
Median [Min, Max]	58.0 [54.0, 87.0]	62.5 [49.0, 89.0]	60.0 [24.0, 90.0]
**TNM_Stage, n (%)**			
0	0 (0%)	0 (0%)	4 (0.6%)
I-II	1 (14.3%)	3 (25.0%)	274 (38.6%)
III-IV	6 (85.7%)	9 (75.0%)	431 (60.8%)
**Smoking History, n (%)**			
No	4 (57.1%)	8 (66.7%)	498 (70.2%)
Yes	3 (42.9%)	4 (33.3%)	211 (29.8%)
**Pathology, n (%)**			
Squamous	1 (14.3%)	0 (0%)	20 (2.8%)
Other	0 (0%)	0 (0%)	1 (0.1%)
Adenocarcinoma	5 (71.4%)	12 (100%)	688 (97.0%)
Missing	1 (14.3%)	0 (0%)	0 (0%)

**Mutation pattern**. Among the 709 *EGFR*-only patients, the frequencies of L858R and exon 19 deletion (19del) were nearly identical (44.4% vs. 42.2%). In contrast, among the seven *EGFR*–*MET*ex14 co-mutant cases, five (71.4%) harbored L858R (Fig. 1A) and two (28.6%) carried 19del, aligning with prior reports showing 75% L858R (*n* = 4) [1, 2]. Notably, four of the five L858R cases acquired *MET*ex14 following EGFR-TKI treatment, whereas one presented with *de novo MET*ex14. Both 19del cases harbored *de novo MET*ex14. *MET* amplification was detected in 57.1% (4/7) of *EGFR*–*MET*ex14 tumors, a markedly higher rate than in *MET*ex14-only cancers (8.3%, 1/12; Fig. 1B) and higher than the ~15% typically reported in *MET*ex14 cohorts [3]. Likewise, earlier reports of *EGFR*–*MET* co-mutants (*n* = 4) noted a *MET* amplification rate of about 50% [1, 2].

**Fig. 1:**
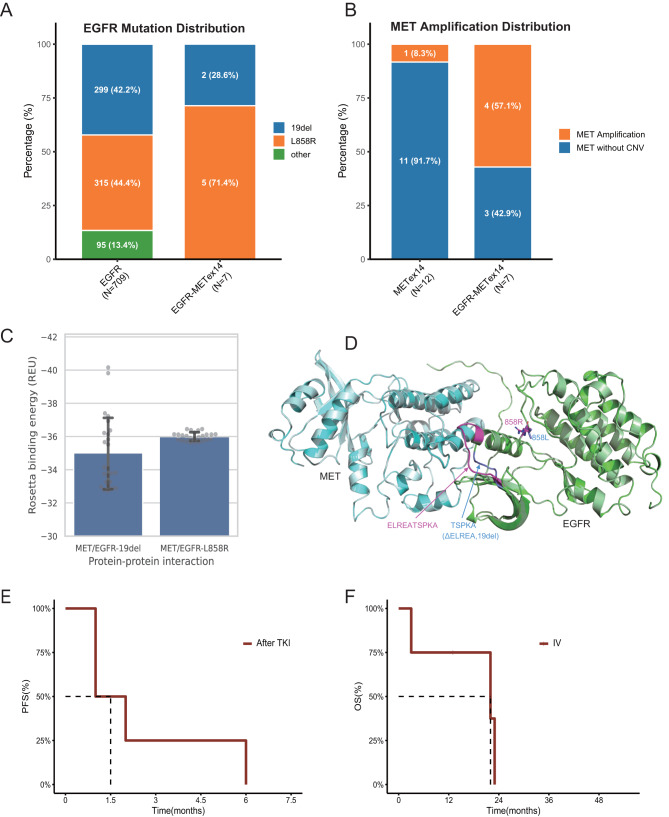
Clinical, molecular and structural features of *EGFR*–*MET*ex14 co-mutated NSCLC versus single-driver cohorts. (**A**) Distribution of *EGFR* mutation subtypes. (**B**) Frequency of *met* amplification. (**C**) Binding energy comparison of MET–EGFR-19del and MET–EGFR-L858R interaction, reported in Rosetta energy units (reu). (**D**) Comparison of structural models of MET–EGFR-19del and MET–EGFR-L858R interactions. MET and EGFR are shown in cyan and green, respectively, for the MET–EGFR-19del model, and in pale cyan and pale green for the MET–EGFR-L858R model. Key residue differences are highlighted in blue (MET–EGFR-19del) and magenta (MET–EGFR-L858R). (**E**) Kaplan–Meier curve of progression-free survival following *MET*ex14 co-mutation in the post-EGFR tki setting. (**F**) Kaplan–Meier curve of overall survival in stage iv co-mutated patients

**Structural modeling.** The EGFR and MET kinase domains are known to interact directly [4], as both domains are located in the cytoplasm. We modeled their kinase domain interactions using comparative modeling in SWISS-MODEL [5], followed by binding energy estimation with Rosetta [6] (Supplementary Methods). Computational analysis suggested that the EGFR-L858R kinase domain bound more strongly to the MET kinase domain than EGFR-19del, as indicated by a lower predicted binding energy (Fig. 1C). This tigher interaction may help explain the predominance of *EGFR*-L858R in the co-mutant cohort. Structural models further supported this difference, with a five–amino acid fragment (ELREA) present at the MET–EGFR-L858R interface but absent from the MET–EGFR-19del complex (Fig. 1D). The loss of this segment likely reduced interdomain contacts and could weakened the binding affinity of EGFR-19del for MET.

**Clinical outcomes**. Prognosis varied by the timing of *MET*ex14 emergence. The four patients who acquired *MET*ex14 under EGFR-TKI pressure deteriorated rapidly despite platinum-pemetrexed chemotherapy or cranial stereotactic radiotherapy, with a median progression-free survival (PFS) of 1.5 months after co-mutation detection (Fig. 1E). None survived beyond six months (Table S1). In contrast, the three *de novo MET*ex14 cases (two *EGFR*-19del and one *EGFR*-L858R) exhibited more heterogeneous outcomes. Among the two stage IV *EGFR*-19del cases, the patient with *MET* amplification progressed rapidly and died within three months, whereas the one without amplification remained progression-free for 13 months on furmonertinib. The remaining stage I *EGFR*-L858R case was recurrence-free five months after lobectomy (Table S1). Notably, *MET* amplification was consistently present in the most rapidly fatal cases but absent in those with durable stability, suggesting an additive oncogenic effect. Across all metastatic co-mutant patients, the median overall survival (OS) was 22 months (Fig. 1F).

**Biological rationale**. *MET*ex14 lacks the Y1003 ubiquitination motif, preventing CBL-mediated degradation and thereby stabilizing the MET protein [7]. Accumulated MET promotes EGFR-MET heterodimerization, activating *AKT*/*ERK* pathways that drive proliferation and survival, and STAT3 signaling that enhances migration and invasion [4]. Computational modeling further suggested that, compared to EGFR-19del, the EGFR-L858R kinase domain exhibited stronger binding affinity for the MET kinase domain, which may contribute to its enrichment among co-mutant tumors and potentially inhibit EGFR-TKI sensitivity. As both *MET*ex14 and *MET* amplification could increase the MET abundance to some extent [7, 8], their co-existence potentially enhance EGFR–MET complex formation and amplify MET-driven oncogenic signaling.

**Therapeutic implications**. Emerging evidence supports dual EGFR/MET blockade in this subset. Suzawa et al. reported an *EGFR*-L858R case with acquired *MET*ex14 plus *MET* amplification that derived more than nine months of benefit from osimertinib plus crizotinib, while sequential monotherapies each failed within three months [1]. Prospective trials of combined EGFR and MET TKIs have shown improved response rates and PFS than EGFR-TKI alone in *MET*-amplified patients [9]. Bispecific antibodies such as amivantamab, which target the extracellular domains of EGFR and MET to intercept ligand-independent heterodimer signaling and prevent MET-driven escape, are being developed. Our findings underscore the importance and relevance of serial plasma or tissue NGS at baseline and disease progression to detect *MET*ex14 early and facilitate enrollment into dual-targeted therapy trials.

**Potential Limitations.** Our study has several limitations. First, the co-mutant sample size was small (*n* = 7) and heterogeneous (e.g., de novo vs. acquired *MET*ex14, presence/absence of *MET* amplification, and varying treatment lines), which may limit the generalizability of our findings. Second, although *EGFR* and *MET* were uniformly and adequately covered, the use of different targeted capture panels may introduce interassay variability. Third, the structural modelling is hypothesis-generating: the EGFR–MET complexes are based on homology models and template-guided superposition (EGFR–HER3 PDB 4RIW; MET kinase PDB 3DKC), yielding static conformations that do not capture dynamics, allostery, post-translational modifications, or membrane context. Finally, treatment decisions in this real-world cohort were influenced by practical considerations such as treatment toxicity, performance status, access, and availability. Some cases were diagnosed in earlier years before dual-target regimens became available in China. Consequently, causal interpretations regarding specific treatment effects should be considered exploratory and require prospective validation.

**Conclusions**. *EGFR*–*MET*ex14 co-mutation is characterized by a predominance of *EGFR*-L858R, frequent *MET* amplification, poor outcomes following on-therapy emergence, and model-supported tighter EGFR-L858R–METex14 heterodimer interactions. These findings support routine molecular re-profiling at progression and the prospective evaluation of dual EGFR/MET-targeted strategies. Given the small cohort size and exploratory design, these conclusions are hypothesis-generating and warrant validation in larger, independent cohorts.

## Electronic supplementary material

Below is the link to the electronic supplementary material.

Supplementary Material 1

## Data Availability

The original contributions presented in the study are included in the article/supplementary material. Further inquiries can be directed to the corresponding author.

## Abbreviations

*EGFR*: epidermal growth-factor receptor
*MET*ex14: *MET* exon 14 skipping
19del: *EGFR* exon 19 deletion
TKI: tyrosine-kinase inhibitor
NGS: next-generation sequencing
PFS: progression-free survival
OS: overall survival
