# Supplementary material for: Clinical and structural insights into concurrent EGFR and MET exon 14 skipping mutations in NSCLC: a multi-center series
Source: Biomark Res. 2025 Nov 28;13:154. doi: 10.1186/s40364-025-00864-1 (PMC12661821; doi:10.1186/s40364-025-00864-1)
Supplement: Supplementary file 1 — Supplementary Material 1 [file 40364_2025_864_MOESM1_ESM.docx]

**Supplementary Materials**

**Methods**

**DNA extraction and targeted NGS**

All patients underwent pathological tissue examination using specimens obtained post-operatively following surgical resection. Tumor tissues were first inspected by pathologists to ensure that they contained at least 20% tumor content and sufficient sample for testing. Genomic DNA from FFPE sections and whole blood control samples were extracted with the QIAamp DNA FFPE Tissue kit and DNeasy Blood and Tissue Kit (Qiagen, USA), respectively. Quantity and quality of the extracted DNA were evaluated by Qubit 3.0 fluorometer and Nanodrop 2000, respectively (Thermo Fisher Scientific). Library preparations were performed using KAPA Hyper Prep kit (KAPA Biosystems) following manufacturer’s protocol. Hybridization-based target enrichment was carried out using the GeneseeqPrime® pan-cancer gene panel with xGen Lockdown Hybridization and Wash Reagents Kit (Integrated DNA Technologies). Captured libraries by Dynabeads M-270 (Life Technologies) were amplified in KAPA HiFi HotStart ReadyMix (KAPA Biosystems) and quantified by qPCR using KAPA Library Quantification Kit (KAPA Biosystems). The target-enriched library was then sequenced on the NextSeq550Dx NGS platform (Illumina) following the manufacturer’s instructions.

**Sequence alignment and data processing**

Trimmomatic was used for FASTQ file quality control. Leading/ trailing low quality (below 20) or N bases were removed. The sequencing data was aligned to the reference Human Genome (hg19) using Burrows-Wheeler Aligner (BWA-mem, v0.7.12). Alignment results underwent de-duplication by Sambamba (PMID: 25697820). Base quality recalibration and indel realignment were processed by Genome Analysis Toolkit (GATK 3.4.0). VarScan2 was employed for calling single-nucleotide variations (SNVs) and insertion/deletions (INDELs) which were identified with a minimum variant allele frequency threshold set at 0.01 and p value threshold for calling variants set at 0.05 to generate Variant Call Format files. All SNVs/indels were annotated with ANNOVAR. The sequencing assay has been validated in compliance with college of American pathologists (CAP) and clinical laboratory improvement amendments (CLIA) with a limit of detection of 1% VAF for tissue. Genomic fusions were identified by FACTERA with default parameters. Copy number variations (CNVs) were detected using CNVkit with default parameters. Depth ratios of above 2.0 (tissue) and below 0.6 were considered as CNV gain and CNV loss, respectively.

**MET-EGFR interaction modeling and binding affinity prediction**

We modeled the interaction between the cytoplasmic kinase domains of MET and EGFR using the following procedure:

First, the MET kinase domain structure (PDB 3DKC, chain A) was superimposed onto the HER3 component (chain A) of the HER3–EGFR heterodimer (PDB 4RIW) using PyMOL, resulting in a hybrid MET–EGFR complex scaffold. It should be noted that this scaffold contains numerous missing residues and may exhibit significant steric clashes at the interface. Additionally, since the EGFR structure in 4RIW is the wildtype, it does not incorporate the EGFR-L858R or EGFR-19del mutations.

Second, the hybrid scaffold was used as a user-defined template to build structural models for the MET–EGFR-19del and MET–EGFR-L858R complexes using SWISS-MODEL, a web-based tool for homology modeling that generates high-quality 3D models of proteins based on their amino acid sequences and known structures of related proteins. Both models showed high quality, with GMQE (Global Model Quality Estimate) scores of 0.88 and QMEANDisCo global scores of 0.81 ± 0.05 for MET–EGFR-L858R and 0.82 ± 0.05 for MET–EGFR-19del. The protein sequences used for modeling are provided in Table S1.

Third, the MET–EGFR-19del and MET–EGFR-L858R models were subjected to local docking refinement using Rosetta (v3.15) with default parameters, generating 500 structural models for each complex. During refinement, Rosetta computed the interaction score (I_sc) for each model. For each complex, the average of the 25 lowest I_sc values was used as the predicted binding affinity, reported in Rosetta energy units (REU).

Based on this approach, the predicted binding affinities for MET–EGFR-19del and MET–EGFR-L858R were –34.972 ± 2.109 REU and –36.002 ± 0.247 REU, respectively, suggesting that MET–EGFR-L858R may have a slightly stronger interaction than MET–EGFR-19del.

| **Table S1. Treatment summary and outcomes for seven NSCLC patients with EGFR-MET co-mutations** | | | | | | | | |
| --- | --- | --- | --- | --- | --- | --- | --- | --- |
| **Patient** | **TNM stage** | **Co-mut type^†^** | **EGFR** | **Treatment Summary** | **PFS ^‡^ (months)** | **PFS**  **status^§^** | **OS (months)** | **OS status^§^** |
| 1 | II→IV | Resistant | L858R | Surgery (July 2016) → Adjuvant chemotherapy/radiotherapy → Recurrence (December 2017), continued chemotherapy → *EGFR*+ detected (August 2020, gefitinib) → Progression (February 2022, *EGFR*/*MET*ex14 + AMP co-mutation) → Palliative therapy (pemetrexed/carboplatin chemo + 15-fraction cranial stereotactic RT) + supportive care → Died (April 2022). | 2 | 1 | 69 | 1 |
| 2 | IV | Resistant | L858R | Gefitinib (August 2021) partial response (PR) → Progression (February 2022) with *EGFR* L858R/T790M → Osimertinib (March 2022) → Stable until October 2022 → Relapse with brain metastases and radiotherapy (January 2023) → MET-driven progression (April 2023) → TP chemotherapy → Admitted (May 2023) with *MET*+*EGFR* co-mutation detected → Died (June 2023). | 1 | 1 | 22 | 1 |
| 3 | IV | Resistant | L858R | Lung adenocarcinoma (IVb) treated with EGFR-TKI → *EGFR* L858R/*MET*ex14 + AMP detected, October 2023) → Metastatic progression → MDT recommended savolitinib, patient discharged → Died (November 2023). | 1 | 1 | NA | 1 |
| 4 | IV | Resistant | L858R | Lung adenocarcinoma (*EGFR* L858R detected, May 2021) → Icotinib initiated (May 2021, achieved PR) → Progressive disease (PD) with metastases (October 2022) & METex14 detected → Died (April 2023). | 6 | 1 | 23 | 1 |
| 5 | IV | Primary | 19del | *EGFR*/*MET* exon 14 skip + AMP detected (July 2021) → Initiated targeted therapy → Discontinued due to severe toxicity → Died (October 2021). | 3 | 1 | 3 | 1 |
| 6 | IV | Primary | 19del | *EGFR*/*MET*ex14 detected (March 2024), furmonertinib (March 2024 – present) + 30 Gy/10-fraction VMAT (March 15-27, 2024) → No toxicity. | 13 | 0 | 13 | 0 |
| 7 | I | Primary | L858R | Surgery (November 11, 2024, Stage I lung adenocarcinoma) → *EGFR* L858R/*MET*ex14 + AMP detected (November 18, 2024) → Ongoing follow-up. | 5 | 0 | 5 | 0 |

Abbreviations: OS, overall survival; PFS, progression-free survival; TNM, Tumor, Node, Metastasis; *MET*ex14, *MET* exon 14 skipping; AMP, *MET* amplification.

^†^Indicates whether the co-mutation is primary (at diagnosis) or associated with EGFR-TKI resistance.

^‡^Time from detection of *EGFR* and *MET* exon 14 skipping co-mutation to disease progression.

^§^0 indicates no event (alive/censored), 1 indicates event occurred (progression or death).

**Table S2. Sequences of MET and EGFR domains used for structural modeling**.

| >MET  TVHIDLSALNPELVQAVQHVVIGPSSLIVHFNEVIGRGHFGCVYHGTLLDNDGKKIHCAVKSLNRITDIGEVSQFLTEGIIMKDFSHPNVLSLLGICLRSEGSPLVVLPYMKHGDLRNFIRNETHNPTVKDLIGFGLQVAKGMKYLASKKFVHRDLAARNCMLDEKFTVKVADFGLARDMYDKEYYSVHNKTGAKLPVKWMALESLQTQKFTTKSDVWSFGVLLWELMTRGAPPYPDVNTFDITVYLLQGRRLLQPEYCPDPLYEVMLKCWHPKAEMRPSFSELVSRISAIFSTFIG  >EGFR-L858R  LVEPLTPSGEAPNQALLRILKETEFKKIKVLGSGAFGTVYKGLWIPEGEKVKIPVAIKELREATSPKANKEILDEAYVMASVDNPHVCRLLGICLTSTVQLITQLMPFGCLLDYVREHKDNIGSQYLLNWCVQIAKGMNYLEDRRLVHRDLAARNVLVKTPQHVKITDFGRAKLLGAEEKEYHAEGGKVPIKWMALESILHRIYTHQSDVWSYGVTVWELMTFGSKPYDGIPASEISSILEKGERLPQPPICTIDVYMIMVKCWMIDADSRPKFRELIIEFSKMARDPQRYLVIQGD  >EGFR-19del  LVEPLTPSGEAPNQALLRILKETEFKKIKVLGSGAFGTVYKGLWIPEGEKVKIPVAIKTSPKANKEILDEAYVMASVDNPHVCRLLGICLTSTVQLITQLMPFGCLLDYVREHKDNIGSQYLLNWCVQIAKGMNYLEDRRLVHRDLAARNVLVKTPQHVKITDFGLAKLLGAEEKEYHAEGGKVPIKWMALESILHRIYTHQSDVWSYGVTVWELMTFGSKPYDGIPASEISSILEKGERLPQPPICTIDVYMIMVKCWMIDADSRPKFRELIIEFSKMARDPQRYLVIQGD |
| --- |
